# Dropout rate from a community-based health insurance scheme and associated factors in the Hagere Mariam district: a mixed method study

**DOI:** 10.3389/fpubh.2025.1514540

**Published:** 2025-09-03

**Authors:** Taye Anbessie Teklemariam, Fitsum Zekarias Mohammed, Getahun Gebre Bogale, Besufekad Mulugeta Urgie, Solomon Hailemeskel Beshah

**Affiliations:** ^1^Department of Public Health, School of Public Health, College of Medicine and Health Science, Debre Berhan University, Debre Berhan, Ethiopia; ^2^Department of Health Informatics, School of Public Health, College of Medicine and Health Science, Debre Berhan University, Debre Berhan, Ethiopia; ^3^Department of Internal Medicine, School of Medicine, College of Medicine and Health Science, Debre Berhan University, Debre Berhan, Ethiopia; ^4^Department of Midwifery, School of Nursing and Midwifery, College of Medicine and Health Science, Debre Berhan University, Debre Berhan, Ethiopia

**Keywords:** community-based health insurance (CBHI), health insurance, universal healthcare coverage (UHC), primary healthcare financing, Ethiopia

## Abstract

**Background:**

Community-based health insurance scheme is an effective strategy to accelerate the progress towards universal health coverage. This scheme plays a crucial role in reducing out-of-pocket expenses for individuals and households, thereby lowering the risk of catastrophic financial consequences. However, high dropout rates, particularly in low-and middle-income countries, continue to pose significant challenges to the successful implementation and continuation of the program. Therefore, understanding the factors contributing to scheme attrition is essential for developing targeted retention strategies and ensuring program effectiveness. Accordingly, this study assessed the dropout rate and associated factors in the Hagere Mariam district, North Shewa, Amhara Region, Ethiopia, in 2024.

**Methods:**

A cross-sectional mixed-method study was conducted to assess community-based health insurance dropout rate and associated factors in the Hagere Mariam district between February 1 and 29, 2024. The quantitative arm involved 634 systematically selected households in the Hagere Mariam district, selected through multistage sampling. The statistical analysis involved a binary logistic regression model, with the community-based health insurance membership status as the dichotomous outcome variable. After initial bi-variable screening (*p* < 0.25), significant variables underwent multivariable analysis, with statistical significance set at *p* < 0.05. The qualitative component employed purposive sampling of 10 key informants and two groups of former community-based health insurance members. The data were then thematically analyzed, and the results were triangulated with the quantitative findings.

**Results:**

The dropout rate from the community-based health insurance scheme among households in the Hagere Mariam district was 19.6% (95% CI = 16.42–22.9%). The poor quality of services (AOR = 7.25; 95% CI: 4.48–11.75), consistent unavailability of prescribed medications (AOR = 2.79; 95% CI: 1.29–6.05), unreimbursed out-of-pocket expenses (AOR = 3.09; 95% CI: 1.19–7.99), and absence of a chronically ill family member in the household (AOR = 3.21; 95% CI: 1.62–6.35) were the factors significantly associated with membership discontinuation.

**Conclusion:**

In the Hagere Mariam district, the dropout rate from the community-based health insurance scheme was significantly higher than the zonal average for the same year. Enhancing service quality through provider training and supervision, ensuring consistent availability of essential medicines, and reforming reimbursement policies to better protect members from financial burdens could significantly improve member retention.

## Introduction

Achieving universal health coverage (UHC) is a critical goal for healthcare systems worldwide, and effective primary healthcare financing is a key strategy to achieve this goal (1, 2). This is especially imperative in low-and middle-income countries (LMIC) where financial barriers frequently hinder access to vital healthcare services (3, 4). Effective primary healthcare financing approaches play a vital role in overcoming these barriers and ensuring that all patients receive the necessary care (5). Among the various healthcare financing approaches, the community-based health insurance scheme (CBHI) has emerged as a pivotal mechanism for advancing UHC, especially in countries with large informal sectors where traditional insurance systems may not be as effective (6, 7). Community-based health insurance models pool resources from individuals who face similar health risks, thereby reducing reliance on out-of-pocket (OOP) expenditures, which are a significant obstacle to accessing vital health care services in many countries, including Ethiopia (4, 6).

Healthcare-related OOP expenses have plunged more than 808 million people worldwide into financial catastrophe, forcing 97 million individuals into extreme poverty (8). A key driver of this crisis is inadequate health insurance coverage, compounded by alarmingly high dropout rates, particularly in low-and middle-income countries (LMICs), leaving households vulnerable to severe financial strain (6, 7).

Alarmingly high dropout rates from health insurance schemes across LMICs have been reported. For instance, Panda et al. in India documented an 80% dropout rate, whereas Van et al. reported a 21.2% dropout rate in Vietnam. Likewise, Emmanuel et al. observed a high dropout rate of 25.1% in Southwestern Uganda, whereas Kagaigai et al. documented an even higher rate of 64% in Tanzania, highlighting significant gaps in financial protection in LMICs (9–12). The healthcare financing situation in Ethiopia is even more concerning. Recent data from the 2019 EDHS reveal that CBHI coverage has plummeted to just 28% of households, a dramatic decrease from 48% in 2016 (13, 14). This troubling trend is further corroborated by multiple regional studies reporting high disenrollment rates in Ethiopia. Research by Kaso et al. in the Gedeo Zone documented a 17.4% dropout rate, while Ashagrie et al. observed an even higher rate of 37% in the Dera District. Similarly, Eseta et al. reported 31.9% disenrollment in the Jimma Zone, and Haile et al. found 21.5% in Arba Minch (14–18).

Community-based health insurance membership discontinuation in Ethiopia is attributed to several factors, including limited knowledge of the scheme, dissatisfaction with healthcare quality, absence of chronically ill family members, high premium costs, failure to reimburse out-of-pocket expenses, large family sizes, long distances to healthcare facilities, and shortages of essential medical supplies (14–18). In addition to these factors, the sociodemographic background of CBHI members, such as age, sex, occupation, educational status, and marital status, has also been found to affect their decision to discontinue membership (14–18).

Andersen’s behavioral model of healthcare service use, prospect theory, principal-agent theory, social capital theory, and the theory of planned behavior can help us better understand how these factors determine healthcare service utilization patterns at different levels.

Andersen’s behavioral model of healthcare service use, for instance, provides a framework for understanding how predisposing factors (household size, education level), enabling resources (income, distance to facilities), and perceived health needs influence service utilization decisions at the individual level (19). This is complemented by the prospect theory, which was first introduced in 1976 by Kahneman and Tversky. This theory elucidates how households in this low-income context evaluate the loss of premium payments as a function of uncertain future health benefits. This calculation often leads to risk-averse decisions to disenroll (20).

The principal-agent theory further examines institutional-level factors, such as critical systemic challenges, particularly information asymmetries between members and providers, coupled with perceived poor service quality at CBHI-contracted facilities, which erode trust in the scheme (21). These theoretical perspectives are further enriched by Putnams’ social capital theory, which examines how variations in traditional risk-sharing mechanisms and trust in local institutions differentially influence CBHI participation (22).

The theory of planned behavior, proposed by Ajzen in 1991, serves as a unifying framework that bridges these levels of analysis by demonstrating how attitudes (perceived value of CBHI), subjective norms (community expectations about enrollment), and perceived behavioral control (confidence in one’s ability to pay the annual fees) collectively shape retention behaviors (23).

In recent years, the drastic increase in the dropout rate from the CBHI program in Ethiopia has led to a significant rise in OOP medical expenditures, which are currently estimated at 22 billion Birr annually (24–26). Consequently, 35% of the population struggles to afford healthcare and often resort to borrowing from relatives or even begging (24–26). Additionally, approximately 10% of individuals are forced to sell their assets to cover exorbitant treatment costs (24–26).

To minimize healthcare-related financial hardship faced by its citizens and accelerate progress toward UHC, the Ethiopian government has devised a comprehensive health financing strategy as part of its Health Sector Transformation Plan (27, 28). A cornerstone of this strategy is the introduction and expansion of the CBHI, which was first piloted in 2011 and later scaled up across the country (24, 28–31). This program aims to alleviate the financial burden of healthcare on individuals and households, particularly those in the informal sector, and improve access to essential healthcare services (24, 29). By 2020, the CBHI had expanded to 827 districts, including all districts in the North Shewa Zone of the Amhara Region (24, 29). However, despite efforts to expand the program, CBHI coverage in Ethiopia remains low, and dropout rates continue to rise sharply. For instance, in the Hagere-Mariam district, the CBHI coverage in 2022 was estimated to be 48%, far below the national target of 85% (29, 32, 33). The district’s dropout rate also surged to 31%, a steep increase from 11% in the previous year, and was considerably greater than the North Shewa Zone average of 5% (33, 34). To make matters worse, the factors behind the surge in dropout rates in the district remain unknown.

Taken together, the high dropout rate from the CBHI scheme in the Hagere Mariam district poses a significant challenge to achieving UHC and providing vulnerable households financial risk protection. Understanding the factors contributing to ever increasing member attrition rate is essential, as persistent attrition undermines the scheme’s financial sustainability and limits access to essential healthcare services. Currently, there is a critical gap in evidence, as no prior studies have specifically examined the factors linked with membership discontinuation in this district, leaving policymakers with limited insights to guide corrective measures. Without a clear assessment of the underlying causes, effective interventions cannot be developed. Therefore, this study is vital not only to identify key factors leading members to drop out of the scheme but also to bridge the existing knowledge gap, providing evidence-based recommendations to improve retention. The findings will inform targeted policy adjustments and program strategies, ensuring the CBHI scheme’s long-term success and equitable healthcare access in the region.

## Methods and materials

### Study area and period

The study was conducted from February 1 to 29, 2024, among CBHI households in the Hagere Mariam district. The district, which is located 130 km from the country’s capital city, Addis Ababa; 643 km from Bahir Dar; and 82 km from Debre Birhan (the zonal capital of the North Shewa Zone), is one of 26 districts in the North Shewa Zone of the Amhara region. It has a mixed warm and cold climate and is divided into 2 urban kebeles and 19 rural kebeles. The district’s total population is estimated at 68,318 with approximately 15,181 households. The healthcare infrastructure includes 4 government health centers, 21 government health posts, and 10 private facilities, including four clinics and six drug stores.

### Study design and population

A community-based cross-sectional design employing both qualitative and quantitative methods was used to assess the dropout rate and associated factors in the Hagere Mariam district. This mixed-methods approach allowed for the concurrent collection of qualitative and quantitative data. The source population for the study comprised all households ever registered for membership in the CBHI scheme within the Hagere Mariam district. The study population comprised households registered for CBHI membership who were selected during the sampling process. However, households registered in the CBHI scheme after January 1, 2023, were excluded from the study. Additionally, the qualitative component of the study involved the purposeful selection of 10 key informants (KIs) and two groups of former CBHI scheme members (a total of 15 individuals).

### Sample size determination

The study sample size was estimated using a single population proportion equation. The adjustments made while calculating the sample size included a 95% confidence interval, a 5% error margin, and a dropout rate of 37.3%, which was taken from a CBHI study conducted in the Dera district, northwestern Ethiopia (18). After further adjustments were made to account for possible nonresponse (17%), the total sample of participants required to accurately estimate the dropout rates in the source population was estimated to be 634. Furthermore, using similar approaches, the required sample sizes for assessing the associated factors were calculated. Ultimately, the largest among the calculated sample sizes was selected, resulting in a final sample size of 634.

### Sampling technique and procedure

Participants in the quantitative study were selected using a multistage sampling technique. First, the district’s 21 kebeles were categorized into 2 urban and 19 rural kebeles. Using a simple random selection technique and following the WHO representation guideline (30–40%), one urban kebele and six rural kebele were chosen. The sample size was then proportionally distributed to the seven kebeles by considering the total number of registered households in each kebele. The district CBHI scheme registration platform was reviewed to obtain a list of registered households. Using this list as the sampling frame, sample households were systematically selected from each kebele. Village leaders and health extension workers helped locate the sampled households within the community.

For the qualitative component, purposive sampling was used to select KIs and focused group discussion (FDG) participants. KIs were chosen based on their expertise and role in the CBHI scheme, and included the CBHI coordinator, medical auditor, and health center heads. The CBHI coordinator and health extension workers facilitated the selection of the KIs. Two groups of FDG participants comprising 7 and 8 former CBHI members were also purposefully selected to be included in the study.

### Operational definitions

**Table:** 

Member	A household head or individual registered for the CBHI who made an annual contribution or whose contribution was covered by another party (7).
Indigent	A household head or individual identified by the Woreda as unable to make annual membership contributions based on specified income criteria (7).
Benefit package	Health services covered by the CBHI scheme (7).
Renewal	Households that registered for the CBHI and paid annual premiums for one year to access the benefit package.
Out-of-pocket payments	Direct medical payments made by CBHI members at the point of care for services covered under the CBHI benefit package.
Full reimbursement of OOP payments	A 100% repayment of eligible out-of-pocket payments for covered healthcare services by the insurance scheme.
Partial reimbursement of OOP payments	Incomplete repayment of eligible out-of-pocket payments for covered healthcare services by the insurance scheme.
No reimbursement of OOP payments	The absence of any repayment for out-of-pocket payments by the insurance scheme.
Dropouts	Households that have not renewed their membership after one year of using the CBHI benefit package (coded as 1 for dropped out and 0 for renewed).
Affordability	The perceived ability to pay the established contribution fee set by the 2020 Amhara regional state directive. A response of “yes” indicates affordability, while “no” indicates unaffordability (35).
Benefit packages	This refers to members’ opinions of the range of services provided by the CBHI scheme, which includes outpatient care, inpatient care, surgical procedures, medical treatments, laboratory tests and diagnostic services.
Quality of service	This refers to members’ evaluations of the quality of services received at healthcare facilities covered by the CBHI scheme, which are rated good, fair, or poor.
Accessibility of health facilities	This is defined as having a healthcare facility within a 2-h walking distance from one. The facility is considered inaccessible if it is beyond this range (36).
Waiting time	The total time a patient spends in the facility from arrival at the registration desk until leaving after the last service. Waiting times of 30 min or less were considered short, whereas waiting times exceeding 30 min were categorized as long (based on the guidelines of the American Institute of Medicine, Jan 25, 2022).

### Data collection methods

Quantitative data were collected using a pretested, interviewer-administered structured questionnaire adapted from previous literature (14–18). Data collection was conducted through face-to-face interviews with household heads after they were provided with detailed information about the study’s purpose. The questionnaire included a section that assessed sociodemographic characteristics, physical access to health institutions, healthcare service utilization, residence characteristics, and CBHI scheme-related factors.

Qualitative data were collected from the KIs and FDG participants. The key informant interviews (KIIs) involved individuals who had expert knowledge of the CBHI scheme, such as the CBHI coordinator, medical auditor, and health center heads. Similarly, FGDs were held with former members of the CBHI scheme. These interviews were open ended, allowing a detailed exploration of the challenges and successes of the CBHI scheme.

### Data quality management

The questionnaires were originally drafted in English, translated into Amharic, and then back-translated into English by a language expert to ensure consistency in the meaning and construction. Three data collectors (two health officers and one MPH holder) and one supervisor (MPH) received 1 day of training on the general concepts of the CBHI, study objectives, interview techniques, data collection tools, procedures, and maintaining participant privacy and confidentiality. The supervisor monitored data collection and provided feedback to the enumerators.

The data collection tools were pretested on 30 randomly selected households from the Angolelatera district, which shares similar demographic and sociocultural features with the study area. Based on the pretest findings, the questionnaire was revised accordingly. During data collection, the principal investigator checked the completed questionnaires for completeness and consistency. The completeness and consistency of the data were also verified during the data entry and cleaning phases.

For the qualitative component, KIIs and FGDs were conducted, where the conversations were recorded using an audio recorder and later transcribed verbatim by a professional transcriptionist. The transcripts were then translated into English by a language expert to ensure accuracy. Thematic analysis was conducted manually, with themes and subthemes derived from the data through an iterative process of coding and categorization. The themes aligned with the study objectives and provided a comprehensive understanding of the factors influencing CBHI dropout rates.

### Data processing and analysis

The quantitative and qualitative data were analyzed separately after simultaneous collection. The principal investigator reviewed the quantitative questionnaires for completeness and consistency. The data were prepared, encoded, and stored in EPI-data version 4.6.0.6, which was subsequently exported to SPSS version 27 for computation. Descriptive statistical methods were used to describe the study participants, and the findings are presented in tables and percentages.

A binary logistic regression model was used to identify significant correlations between membership status and several independent variables. The *p*-value cutoff points of ≤0.25 and <0.05 were used during the bi-variable and multivariable regression analyses, respectively. The Hosmer–Lemeshow test was used to examine the model’s fitness and sample adequacy, with the result indicating a good fit (0.719). The presence of multicollinearity between variables was also checked using the variance inflation factor (VIF), with a maximum VIF of 1.872 indicating no multicollinearity. The strength of the association between dropping out of the CBHI scheme and explanatory variables was ultimately presented as AORs calculated at the 95% CI and 5% margin of error.

The audio recordings and notes from the interviews were transcribed verbatim and read multiple times to ensure clarity. Themes were developed from the text data after thorough reading, and the data were coded according to the identified key themes. Thematic analysis was conducted manually. The qualitative findings were presented narratively alongside the quantitative data through triangulation, with selected quotes supporting the quantitative findings to strengthen the overall study.

## Results

### Sociodemographic characteristics of the participants

This study included 616 households, and the response rate was 97.2%. Participants had a mean age of 45 years (SD ± 13.18), ranging from 20 to 77 years. The sample revealed a significant gender disparity, with males constituting 83% of the respondents. The average household size was 4.5 members (SD ± 1.12), reflecting typical family structures in the region. The residential distribution revealed that 86.9% lived in rural areas, whereas only 13.1% resided in urban settings, consistent with Ethiopia’s predominantly rural population distribution. The majority of participants (83.4%) were married, 7.5% were widowed, and the remaining were either single or divorced. Religious affiliation was nearly unanimous, with 99.4% of respondents identifying themselves as Orthodox Christians. Occupational data indicate that 96.6% of respondents were farmers, reflecting the agricultural basis of the local economy. The ethnic composition was predominantly Amhara (91.1%), with a small representation from Oromo and other ethnic groups. Educational attainment was limited, with 55.7% receiving no formal education (Table 1).

**Table 1:** Socio-demographic characteristics of the respondents in Hagere Mariam district, North Shewa, Amhara, Ethiopia, 2024.

Variables	Category	CBHI status		Total, *N* (%)
		Dropped, *n* (%)	Renewed, *n* (%)	
Sex	Male	104 (20.4)	407 (79.6)	511 (83)
	Female	17 (16.2)	88 (83.8)	105 (17)
Age	20–29	18 (22)	64 (78)	82 (13.3)
	30–39	35 (24)	111 (76)	146 (23.7)
	40–49	31 (18)	141 (82)	172 (27.9)
	≥50	37 (17)	179 (83)	216 (35.1)
Religion	Orthodox	120 (19.6)	492 (80.4)	612 (99.4)
	Others	1 (25)	3 (75)	4 (0.6)
Occupation	Farmers	118 (19.8)	477 (80.2)	595 (96.6)
	Merchant	2 (13.3)	13 (86.7)	15 (2.4)
	Daily laborer	1 (16.6)	5 (83.4)	6 (1)
Marital status	Married	108 (20.8)	412 (79.2)	520 (84.4)
	Others	13 (13.5)	83 (86.5)	96 (15.6)
Ethnicity	Amhara	112 (20)	449 (80)	561 (91.1)
	Others	9 (16.4)	46 (83.6)	55 (8.9)
Educational status	Unable to read and write	30 (16.7)	156 (83.3)	186 (30.2)
	Can only read and write	27 (17.2)	130 (82.8)	157 (25.5)
	Primary	57 (26.9)	155 (73.1)	212 (34.4)
	Secondary and higher	7 (11.5)	54 (88.5)	61 (9.9)
Residence	Rural	104 (19)	431 (81)	535 (86.9)
	Urban	17 (21)	64 (79)	81 (13.1)

### Community-based health insurance scheme-related factors

Among the surveyed households, nearly half reported purchasing medications or undergoing laboratory tests at private health institutions. Of these, only 21.4% received full reimbursement for their OOP expenses, whereas 27% received no reimbursement at all. Regarding the cost of services, almost all participants (96.4%) considered the CBHI scheme’s pre-set premium pricing to be reasonable, with 89% explicitly confirming that they are able to afford it. Furthermore, 94% of respondents reported that the benefit package offered was adequate (Table 2).

**Table 2:** Community-based health insurance-related factors among the respondents in the Hagere Mariam district, North Shewa, Amhara, Ethiopia, 2024.

Variables	Category	CBHI status		Total, *n* (%)
		Dropped, *n* (%)	Renewed, *n* (%)	
Reimbursement	Fully reimbursed	9 (13.8)	56 (86.2)	65 (10.6)
	Partially reimbursed	41 (26.1)	116 (73.9)	157 (25.5)
	Not reimbursed at all	32 (36.8)	55 (63.2)	87 (14.1)
	Did not receive any services from private institutions	39 (12.7)	268 (87.3)	307 (49.8)
Premium packages	Affordable	116 (19.5)	478 (80.5)	594 (96.4)
	Not affordable	5 (22.7)	17 (77.3)	22 (3.6)
Able to pay for premium	Yes	106 (87.6)	442 (89.3)	548 (89)
	No	15 (12.4)	53 (10.7)	68 (11)
Benefit package	Enough	109 (18.8)	470 (81.2)	579 (94)
	Not enough	12 (32.4)	25 (67.6)	37 (6)

### Qualitative findings

All participants in the FGDs agreed that the current reimbursement system under the CBHI program is flawed. A 48-year-old ex-member described the situation as follows: “*After my child became unwell, I went to the closest public hospital for treatment. The medication I was given by the doctor on duty was not stocked at the hospital’s pharmacy. Subsequently, I bought the medications at a private pharmacy for 8,000 ETB. The CBHI office reimbursed only 400 ETB after the receipts for reimbursement were submitted. I inquired about the discrepancy and was informed that reimbursement amounts are determined by government-specified health facility prices rather than the true costs at private pharmacies. The discrepancy prompted me to terminate my enrollment in the health insurance plan.*”

The KIIs have highlighted members’ frequent grievances concerning the reimbursement process, with a 42-year-old KI, who was a medical auditor at the time, commenting that many members are uninformed about the reimbursement standards, resulting in the denial of their claims. “*Members anticipate receiving reimbursement for expenses incurred at private establishments based on actual costs, however, policy permits reimbursement only based on prices established by the government. The absence of clarity and transparency leads to member dissatisfaction, which may cause them to discontinue their participation in the program.*”

The participants generally expressed satisfaction with the services offered by the benefit package; however, in-depth interviews revealed some problematic issues, as highlighted by a 38-year-old CBHI coordinator. “*On paper, the benefit package seems sufficient, but in reality, numerous promised healthcare services are inaccessible in most hospitals.*”

### Accessibility, utilization, and quality of services

The study revealed that 99.2% of the participants could reach the nearest healthcare center within 2 h. However, hospital access was markedly poorer, with 71.4% of the respondents needing to travel over 2 hours to reach the nearest hospital. In terms of waiting time, more than three quarters (84%) of the respondents were able to receive treatment within 30 min of admission. However, significant concerns emerged regarding the reliability of service, with only 65.3 and 77.1% of respondents being able to consistently access the prescribed medications and laboratory tests at CBHI-covered facilities. However, despite these challenges, 80.2% of respondents regard overall service quality as good (Table 3).

**Table 3:** Accessibility, utilization, and quality of healthcare services in Hagere Mariam district, North Shewa, Amhara, Ethiopia, 2024.

Variables	Category	CBHI Status		Total *N* (%)
		Dropped, *n* (%)	Renewed, *n* (%)	
Travel time to the nearest health center	<2 h	115 (19.4)	478 (80.6)	593 (96.3)
	>2 h	6 (26)	17 (74)	23 (3.7)
Travel time to the nearest hospital	<2 h	35 (20)	141 (80)	176 (28.6)
	>2 h	86 (19.5)	354 (80.5)	440 (71.4)
Availability of prescribed medication	Always available	59 (14.7)	343 (85.3)	402 (65.3)
	Sometimes not available	43 (25.9)	123 (74.1)	166 (27)
	Always not available	19 (39.6)	29 (60.4)	48 (7.7)
Availability of prescribed laboratory test	Always available	87 (18.3)	388 (81.7)	475 (77.1)
	Sometimes not available	28 (23.3)	99 (76.7)	127 (20.6)
	Always not available	6 (42.8)	8 (57.2)	14 (2.3)
Waiting time	≤30 min	100 (19.3)	418 (80.7)	518 (84.1)
	>30 min	21 (21.4)	77 (78.6)	98 (15.9)
Accessibility	<2 h	98 (15.2)	428 (84.8)	526 (85.4)
	≥2 h	23 (25.6)	67 (74.4)	90 (14.6)
Quality of service	Good	59 (11.9)	435 (88.1)	494 (80.2)
	Poor	62 (50.8)	60 (49.2)	122 (19.8)

### Qualitative findings

Similar to the descriptive reports, concerns regarding access to healthcare facilities were consistently reported in the FGDs, in which participant’s uniformly highlighted difficulties in accessing hospitals. As one participant, aged 58, noted: *“Our district has a health center located roughly 2 hours away and a private clinic that is even closer, yet unfortunately, there are no hospitals in the area. Those requiring advanced care had to travel to either Debre Berhan or Minjar. Access to healthcare could be significantly improved by a district hospital.”*

The qualitative enquires also revealed analogous service availability issues, with key stakeholders noting the challenges members encounter in accessing most services. A 35-year-old head of a health center shaded light into the systemic challenges saying*: “Our health center commonly experience shortage of medical supplies, largely due to delays from government suppliers. As a result, most patients are forced to purchase their medications at private pharmacies. This not only increases out-of-pocket expenses but also undermines patients’ trust in the CBHI scheme.”*

Data from descriptive reports indicate that over three-quarters of the respondents expressed satisfaction with the overall services, yet qualitative analysis has highlighted significant concerns regarding service quality. A 46-year-old participant in a focus group discussion recalled his personal experiences. *“Two years ago, I visited the nearest health center because I was not feeling well. The healthcare provider available at the time I was diagnosed with gastritis and prescribed medications. However, after taking them for a week, I still did not feel better. Therefore, I decided to seek a second opinion at another hospital, where an ultrasound-aided examination revealed that the issue was actually intestinal, requiring me to undergo major surgery to recover. Experiences like this make me question the quality of services at health centers.”*

### Household characteristics

In nearly one-quarter (23.7%) of households, at least one member was chronically ill. Regarding family structure, most households (62.5%) accommodated no children younger than 5 years of age, and the majority (82.1%) consisted of five or fewer family members (Table 4).

**Table 4:** Characteristics of the households in the Hagere Mariam district, North Shewa, Amhara, Ethiopia, 2024.

Variables	Category	CBHI status		Total, *N* (%)
		Dropped, *N* (%)	Renewed, *N* (%)	
Chronic illness	Yes	12 (8.2)	134 (91.8)	146 (23.7)
	No	109 (23.2)	361 (76.8)	470 (76.3)
Illness within the last 3 months	Yes	21 (14.3)	126 (83.7)	147 (23.9)
	No	100 (21.3)	369 (78.7)	469 (76.1)
Family size	≤5	98 (19.3)	408 (80.7)	506 (82.1)
	>5	23 (21)	87 (79)	110 (17.9)
Older adults (age ≥60)	Yes	19 (16)	99 (84)	118 (19.2)
	No	102 (20.5)	396 (79.5)	498 (80.8)
Children <5 years	Yes	39 (16.9)	192 (83.1)	231 (37.5)
	No	82 (21.3)	303 (78.7)	385 (62.5)
Length of enrollment	1–3 years	95 (20)	406 (80)	501 (81.3)
	≥4 years	26 (22.6)	89 (77.4)	115 (18.7)

### Dropout rates from community-based health insurance schemes

**Figure 1:**
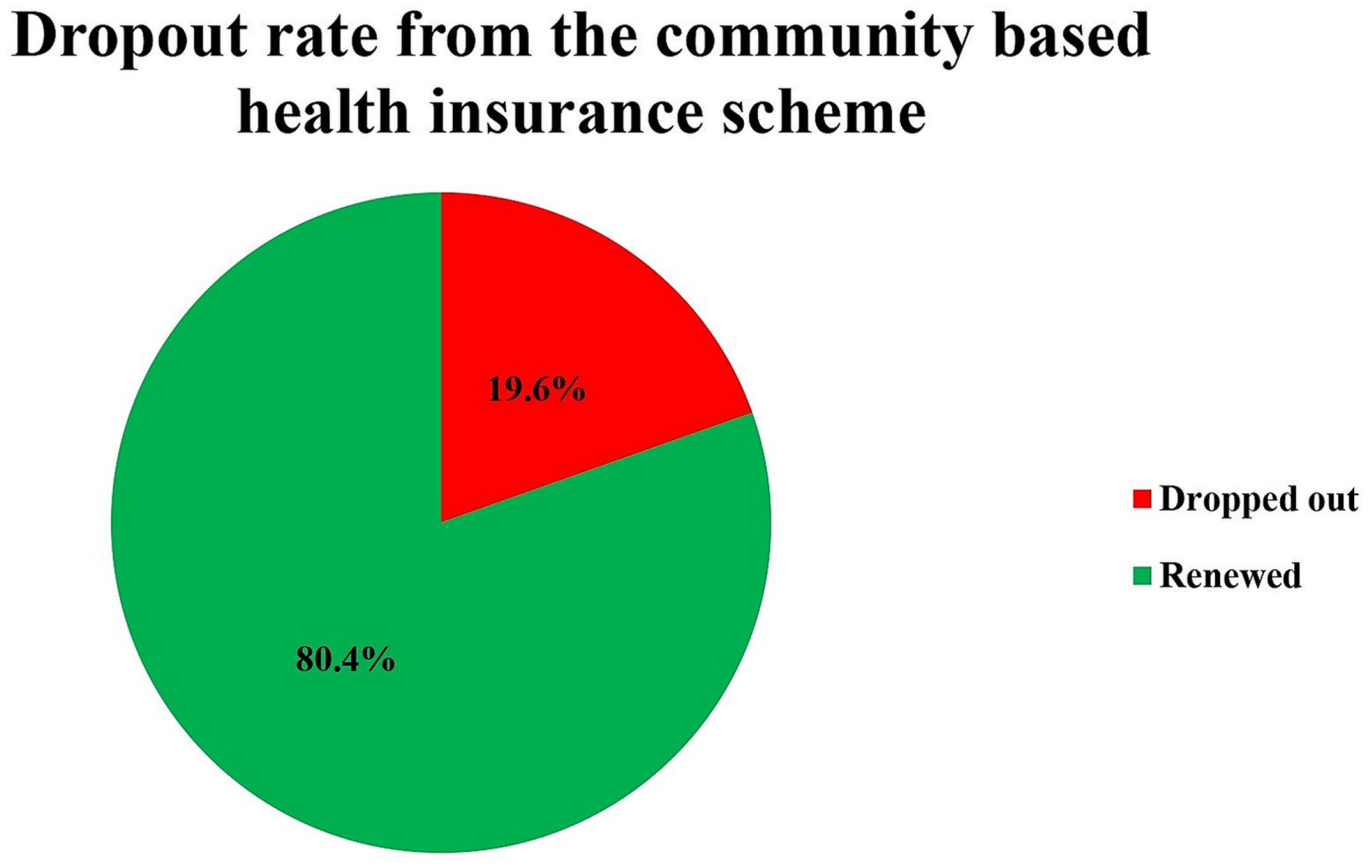
Dropout rate from the community-based health insurance schemes in the Hagere Mariam district, North Shewa, Amhara, Ethiopia, 2024.

The dropout rate from the CBHI scheme in the Hagere Mariam district was 19.6% (95% CI = 16.4–22.9%), with 121 households discontinuing their membership (Figure 1).

### Factors associated with community-based health insurance membership

The preliminary bivariable analysis identified several variables significantly associated with the dropping out at *p* ≤ 0.25. These factors comprised household characteristics (presence of chronically ill family member or children under the age of 5 years), financial protection aspects (reimbursement of OOP expenses), and members’ opinion regarding service quality, medication/laboratory test availability, and adequacy of benefit packages. All identified variables were subsequently entered into a multivariable logistic regression model for further analysis, with *p* value set at <0.05.

The multivariate logistic regression analysis showed that households without a chronically ill family member had 3.21 times higher odds of dropping out of the CBHI scheme than those with a chronically ill member (AOR = 3.21, 95% CI = 1.62–6.35). Similarly, households that perceived the quality of care as poor had 7.25 times higher odds of discontinuing the scheme than those who perceived it as good (AOR = 7.25, 95% CI = 4.48–11.75).

The study also found that medication availability significantly influenced retention. Households that frequently experienced unavailability of prescribed medications at insured facilities had 2.79 times higher odds of dropping out than those with consistent access (AOR = 2.79, 95% CI = 1.29–6.05). Finally, households without reimbursement for out-of-pocket (OOP) expenses had 3.09 times higher odds of disenrollment than those who received full reimbursement (AOR = 3.09, 95% CI = 1.19–7.99) (Table 5).

**Table 5:** Binary logistic regression analysis of factors associated with community-based health insurance membership in Hagere Mariam district, North Shewa, Amhara, Ethiopia, 2024.

Variable		CBHI status		COR (at 95%CI)	AOR (at 95%CI)	*p*-value
		Dropped (%)	Renewed (%)			
Availability of medications	Always available	59 (14.7)	343 (85.3)	1	1	
	Sometimes not available	43 (25.9)	123 (74.1)	2.03 (1.30–3.17)	1.71 (0.94–3.1)	0.77
	Always not available	19 (39.6)	29 (60.4)	3.81 (2.01–7.23)	2.82 (1.3–6.12)*	0.009
Availability of laboratory tests	Always available	87 (18.3)	388 (81.7)	1	1	
	Sometimes not available	28 (23.3)	99 (76.7)	1.35 (0.85–2.16)	0.72 (0.39–1.34)	0.30
	Always not available	6 (42.8)	8 (57.2)	2.23 (0.66–7.57)	1.76 (0.5–6.2)	0.38
Accessibility of health facility	Accessible	98 (15.2)	428 (84.8)	1	1	
	Not accessible	23 (25.6)	67 (74.4)	1.45 (0.89–2.53)	1.63 (0.89–2.96)	0.11
Chronic illness	Yes	12 (8.2)	134 (91.8)	1		
	No	109 (23.2)	361 (76.8)	3.37 (1.79–6.32)	3.17 (1.6–6.27)*	<0.001
Quality of health services	Good	59 (11.9)	435 (88.1)	1	1	
	Poor	62 (50.8)	60 (49.2)	7.62 (4.87–11.91)	7.27 (4.48–11.79)*	<0.001
Benefit package	Yes	109 (18.8)	470 (81.2)	1	1	
	No	12 (32.4)	25 (67.6)	2.07 (1.01–4.25)	0.98 (0.42–2.41)	0.99
OOP reimbursement	Fully reimbursed	9 (13.8)	56 (86.2)	1	1	
	Partially reimbursed	41 (26.1)	116 (73.9)	2.19 (0.99–4.84)	2.19 (0.88–5.44)	0.90
	Not reimbursed at all	32 (36.8)	55 (63.2)	3.62 (1.58–8.28)	2.97 (1.15–7.71)*	0.025
	Not bought from private	39 (12.3)	268 (87.3)	0.91 (0.42–1.98)	1.08 (0.46–2.55)	0.86
Children <5 years	Yes	39 (16.9)	192 (83.1)	1	1	
	No	82 (21.3)	303 (78.7)	1.33 (0.87–2.03)	1.59 (0.98–2.58)	0.59

* Statistically significant; 1 = reference group.

### Qualitative findings

A 40-year-old CBHI coordinator shared her expert observation regarding the factors that lead members to discontinue or renew their membership: *“Members that renew their CBHI membership tend to have larger families, a history of referrals for treatment, chronic illnesses, or recent hospital follow-ups. The coordinator explained the reasons for renewing membership as members with those characteristics perceive greater value in the scheme due to their frequent healthcare needs. On the other hand, the coordinator added, members who discontinue their membership are typically those with infrequent illnesses, whose medical expenses are not reimbursed, or who are dissatisfied with the quality of services provided.”*

## Discussion

This study examined the dropout rate and factors associated with CBHI membership in the Hagere Mariam district of North Shewa, Amhara Region, Ethiopia. The findings revealed that 19.6% (95% CI: 16.4–22.9%) of CBHI members in the Hagere Mariam district discontinued their membership between the time of the scheme introduction in 2011 and 2023.

In line with the dropout rate observed in the Hagere Mariam district, studies by Haile et al. and Kaso et al. also recorded a dropout rate of 21.5% in the Arba Minch survey site and 17.4% in Gedeo Zone, in Ethiopia, respectively (15, 17). However, the dropout rate observed in the Hagere Mariam district was significantly lower than the rates recorded in the Dera District (37% according to Ashagrie et al.) and in the Manna District of Jimma Zone, which was 31.9%, as reported by Eseta et al. (14, 18). In a wider comparison, the dropout rate in Hagere Mariam district was much higher than the national (9.5%) and Zonal (4.5%) average dropout rates for the same calendar year (34).

The comparative analysis of CBHI dropout rates between the Hagere Mariam district and other global contexts yields important insights. While Emmanuel et al. reported a moderately higher dropout rate of 25.1% in Southwestern Uganda; Kagaigai et al. documented a substantially elevated rate of 64% in Tanzania, more than triple the Hagere Mariam district’s observed rate (11, 12). This pattern of variation continues in Asian contexts, where Panda et al. identified an exceptionally high 80% dropout rate in rural India, contrasting sharply with the more comparable 21.2% rate found by Van et al. in Vietnam (9, 10).

The observed variations in CBHI dropout rates across different settings can be attributed to several key factors. Differences in population characteristics, particularly between urban and rural settings and across income levels, may explain some of the divergence in the findings. Methodological approaches, including sampling strategies and study timelines, could further contribute to measuring disparities. Underlying socioeconomic conditions such as educational attainment and healthcare accessibility patterns appear to play significant roles, while temporal factors like policy reforms and economic fluctuations may introduce period-specific effects.

Key factors influencing CBHI members’ decision to leave or maintain their membership were found to include the standard of healthcare services provided, the presence of long-term illnesses within the family, reimbursement of out-of-pocket expenses, and access to prescribed medications at healthcare facilities covered under the plan.

Household dissatisfaction with the service quality at CBHI-contracted facilities significantly increased the odds of membership discontinuation. This finding demonstrates robust cross-regional consistency, which is consistent with the findings of Conde et al. (West Africa), Nshakira-Rukundo et al. (Uganda), Eseta et al. (Mana District), Kaso et al. (Gedeo Zone), Haile et al. (Arba Minch), and Demissie et al. (Northwest Ethiopia) (11, 14, 15, 17, 37, 38). The convergence of these findings across diverse geographical contexts suggests that CBHI members universally expect and actively seek quality healthcare services. When contracted facilities fail to meet these expectations, the resultant dissatisfaction frequently precipitates membership termination as beneficiaries pursue alternative healthcare financing options. This finding underscores that service quality is a critical determinant of CBHI sustainability because it directly influences patient satisfaction, program loyalty, and the perceived value of insurance coverage. The consistent replication of this finding across multiple studies indicates that service quality represents a fundamental programmatic requirement rather than a context-specific preference.

The availability of prescribed medications at CBHI-contracted health facilities emerged as another critical element in membership retention. Households unable to consistently obtain prescribed medications at insured facilities had significantly higher odds of dropping out than those with reliable access. The key informant and focused group discussions further revealed that medication shortages frequently compelled patients to procure essential drugs from private pharmacies at substantially higher costs. This finding is consistent with studies conducted by Halie et al. in Arbaminch, Zepre et al. in Gurage Zone, and Acharya et al. in Lumbini Province, Nepal (15, 39, 40). The convergence of these findings across diverse settings suggests a systemic challenge: medication stockouts not only create patient inconvenience but also impose additional financial burdens, thereby eroding confidence in CBHI-covered services. When compounded by frequent rejections of OOP expense reimbursements, as discussed earlier, this dissatisfaction creates a strong disincentive for scheme continuation, ultimately contributing to member attrition.

Households without a chronically ill family member were at higher odds of dropping out of the scheme compared to those with chronically ill members. This finding is consistent with studies conducted by Demissie et al. in Northwestern Ethiopia and by Eseta et al. in the Mana District of Jimma Zone (14, 38). A plausible explanation for this trend is the perceived utility of insurance coverage. Households lacking chronically ill members may undervalue CBHI retention because of relatively infrequent healthcare needs. In contrast, households with chronically ill individuals who face higher medical expenses from regular treatments demonstrate greater incentive to sustain membership. This pattern reflects adverse selection in which individuals with elevated healthcare demands are more likely to enroll in and maintain insurance coverage. This phenomenon may be explained by the fact that households without chronically ill members perceive less value in maintaining membership due to infrequent healthcare needs. Conversely, households with chronically ill members tend to retain their membership due to the high cost of regular healthcare visits. This reflects a form of adverse selection in which individuals with greater healthcare needs are more likely to join and remain in the CBHI scheme.

Last but not least, the odds of dropping out were found to be higher among households in which OOP expenses were not reimbursed than among households in which OOP expenses were reimbursed. The qualitative findings further revealed widespread dissatisfaction among the members, primarily due to unclear OOP reimbursement policies. A key point of contention was that reimbursements were calculated on the basis of government-set prices rather than actual incurred costs. This finding aligns with the work of Demissie et al. (38), who observed similar patterns in a study conducted in the north western region of Ethiopia. A contributing factor to these shared observations may be the lack of awareness among the surveyed members regarding reimbursement criteria. This knowledge gap frequently led to rejection of OOP reimbursement claims, thereby fostering dissatisfaction with the CBHI scheme.

## Strengths and limitations of the study

The strength of this study is its use of a mixed-methods approach, in which quantitative data are combined with qualitative findings. This combination enhances the depth and richness of the information. Additionally, the study was conducted in both rural and urban settings, thereby generating comprehensive insights into the problem.

Despite the aforementioned strengths, the study was limited by its single study area, which may have undermined the generalizability of the findings to other areas. Furthermore, potential biases such as selection bias, knowledge bias, and recall bias may have affected the results, particularly regarding participant responses.

## Conclusion

This study revealed that the dropout rate from the CBHI in the Hagere Mariam district is significantly higher than the zonal rate. Key factors causing CBHI members to dropout included poor service quality, the absence of chronic illness within households, unreimbursed OOP expenses, and the unavailability of prescribed medications at government health facilities.

Based on the findings, enhancing service quality through provider training and supervision could significantly improve member retention in the district. In addition, ensuring consistent availability of essential medical supplies at contracted healthcare facilities is another critical factor in reducing the OOP expenses and improve member retention. To further alleviate financial burdens, reimbursement policies should be revised and improved to offer better protection against, OOP expenses. Establishing model community pharmacies and forming contractual agreements with organizations like the Red Cross could also help reduce OOP costs by ensuring access to affordable medicines and supplies for CBHI members.

## Data Availability

The raw data supporting the conclusions of this article will be made available by the authors, without undue reservation.
